# HIV integration and T cell death: additional commentary

**DOI:** 10.1186/1742-4690-10-150

**Published:** 2013-12-09

**Authors:** Arik Cooper, Mayra García, Constantinos Petrovas, Takuya Yamamoto, Richard A Koup, Gary J Nabel

**Affiliations:** 1 Virology Laboratory, Vaccine Research Center, National Institute for Allergy and Infectious Diseases, National Institutes of Health, Bldg. 40, Room 4502, MSC-3005, 40 Convent Drive, Bethesda, MD 20892-3005, USA; 2 Immunology Laboratory, Vaccine Research Center, National Institute for Allergy and Infectious Diseases, National Institutes of Health, Bldg. 40, Room 4502, MSC-3005, 40 Convent Drive, Bethesda, MD 20892-3005, USA; 3 Current address: Sanofi, 640 Memorial Drive, Cambridge, MA 02139, USA

## Abstract

Estaquier et al. provide commentary on our paper that elucidated the mechanism by which HIV-1 causes cell death in activated CD4 T lymphocytes. We showed that proviral DNA integration triggers DNA-PK dependent death signaling, leading to p53 phosphorylation and cell demise (Cooper A et al. *Nature***498:**376-379, 2013). They have raised several hypothetical points that we further clarify here.

## 

1. In their commentary, the authors note that cell death in our study was observed only in cells lacking p24 antigen expression (p24^-^) and argue that a mechanism in which integration triggers death would lead to cell killing before the virus had a chance to replicate in its target cell.

Response: Estaquier et al. provide no data to support their claim that cell death is immediate, and none was suggested in the paper. In our study, we showed that viral gene expression can be detected prior to the onset of cell death using a GFP reporter gene. Thus, we noted that dying p24^-^ cells originate from productively infected cells that express viral genes before they lose expression, which is concomitant with cell death (Figure two c in ref. [1]). The explanation for the loss of viral proteins upon cell death is unknown but may result from activation of cellular proteases that degrade them or from epigenetic downregulation. This finding explains why death is observed only in p24^-^ cells and indicates that the virus productively infects its host cell prior to inducing death, a notion reinforced by the observed viral spread in primary cultures infected with a replication-competent HIV-1(Figure one c in ref. [1]). We found that productively infected cells die within 2 to 4 days after infection *in vitro* and within 3 days *ex vivo*, consistent with the *in vivo* half-life of CD4 lymphocytes in humans infected with HIV-1 and in macaques infected with SIV [2,3]. Given that HIV-1 integrates its cDNA within 24 hours following infection [4,5], the onset of cell death is relatively delayed, possibly as a result of anti-apoptotic activities of host and viral machineries [6-8], allowing HIV-1 sufficient time to complete its replication cycle. The DNAPK-p53 death pathway is therefore induced in productively infected cells and can be regarded as an innate defense mechanism that eliminates them. HIV-1 replication is reduced but not abolished by this death response [1,9].

2. Estaquier et al. cite previous reports concerning the role of DNA-PK in lentiviral replication and suggest that this enzyme may have a protective role against cytotoxic effects exerted by high MOI infections.

Response: Previous studies used transformed cell lines rather than primary T cells, and lentiviral vectors rather than replication-competent virus. In our report, we examined the roles of DNA-PK in a physiological context using low MOI infections of a replication-competent HIV-1 in activated primary CD4 T cells, the relevant HIV-1 cell target. Also, the previous studies used a knockout approach, whereas we used highly specific pharmacological inhibitors of this kinase. Because DNA-PK has additional, non-catalytic roles in DNA damage repair [10], knockdown may exert effects on cells not observed with the chemical drug, which only inhibits the catalytic kinase activity. Our findings are consistent with growing evidence that DNA-PK plays a role in cell death induction under a range of physiological conditions [10-12]. Notably, CD4 T-cell activation, which is strongly associated with HIV-induced CD4 lymphocyte depletion *in vivo*[13], promotes nuclear translocation and activation of DNA-PK [14], providing further support for its proapoptotic function during HIV-1 infection.

3. The authors question whether our findings are relevant to lentiviral infection *in vivo*, given the low frequency of CD4 T cells infected with HIV-1 during chronic infection [15]. They also raise concerns regarding the interpretation of our *ex vivo* experiments, arguing the proportion of cell death observed in these experiments seemed too high relative to the CD4 cell counts measured in the patients from which the samples had been received.

Response: The paper did not directly investigate nor make definitive claims regarding *in vivo* effects, though it generated testable hypotheses that were discussed. The comments from Estaquier therefore go beyond the scope of the paper and are speculative. The potential relevance of the paper is supported by several *in vivo* studies that have unequivocally demonstrated that during the early, acute stage of SIV infection, 30-60% of the activated memory CD4 T cells throughout the body are productively infected and eliminated within days of infection as a result of a direct viral cytopathic effect [3,16,17]. Similarly, studies in human subjects have shown that activated CD4 lymphocytes, the major targets for HIV-1 infection, are massively depleted during the acute phase, largely as a result of cytopathic effects associated with productive HIV-1 infection [13,18-20]. Our study showing that integration triggers a death signal in the course of productive infection in activated CD4 T cells is consistent with these previous analyses, and it may therefore be relevant to *in vivo* infection. This relevance is further demonstrated by our *ex vivo* experiments in which, following cell activation, profound death of CD4 T cells was observed in samples from untreated HIV-1-infected subjects but not from healthy donors. A substantial proportion of dying cells harbored viral DNA, and cell death was largely alleviated by raltegravir, establishing a causal link between viral integration and cell death *ex vivo*. Activation sensitizes cells to additional rounds of viral replication and the consequent cell death, providing the likely explanation for the massive death observed *ex vivo* even in samples taken from patients with a relatively high CD4 T cell count. Additional mechanisms such as activation-induced cell death and CTL-mediated cytotoxicity may have contributed to activated CD4 T cell depletion *ex vivo*, because cell death was not completely blocked by the antiretroviral drugs and not all dying cells harbored viral DNA. Collectively, we suggest that the mechanism described in our study drives loss of activated CD4 lymphocytes productively infected with HIV-1, and additional mechanisms may contribute to CD4 depletion of uninfected as well as abortively infected cells [21-23].

4. Estaquier et al. suggest that our study claims that every cell integrating viral DNA automatically dies and suggest that such model would be inconsistent with non-pathogenic SIV infection, in which less cell death has been documented despite sustained viral replication.

Response: We do not suggest that every cell dies after infection. In fact, our data suggests that only activated T cells are susceptible to this mechanism of cytolysis, a central theme of the paper. The question of pathogenic versus non-pathogenic SIV infections is beyond the scope of our study, as we have not experimentally addressed them. Nevertheless, massive depletion of CD4 lymphocytes from the gastrointestinal tract was reported during the acute phase of both pathogenic and non-pathogenic SIV infections, suggesting that in both cases target cells are susceptible to direct lysis by the virus [24]. Cell death by the mechanism described in our study is therefore not inconsistent with non-pathogenic SIV infections *in vivo*. We agree with the authors that non-pathogenic SIV infections are associated with lower activation of CD4 lymphocytes, potentially providing protection from activation-induced cell death [24-26]. Interestingly, nuclear localization and activity of DNA-PK correlate with CD4 T cell activation and are regulated during the cell cycle [14,27]. These studies therefore suggest that proviral DNA integration does not automatically lead to cell death and that the cytopathic effects triggered by viral integration may also be mitigated in the course of non-pathogenic SIV infection.

## Competing interests

The authors declare that they have no competing interests.
